# Cross-Modal Sensory Integration of Visual-Tactile Motion Information: Instrument Design and Human Psychophysics

**DOI:** 10.3390/s130607212

**Published:** 2013-05-31

**Authors:** Yu-Cheng Pei, Ting-Yu Chang, Tsung-Chi Lee, Sudipta Saha, Hsin-Yi Lai, Manuel Gomez-Ramirez, Shih-Wei Chou, Alice M. K. Wong

**Affiliations:** 1 Department of Physical Medicine and Rehabilitation, Chang Gung Memorial Hospital at Linkou, No. 5, Fushing St, Taoyuan 333, Taiwan; E-Mails: taiwan.changty@gmail.com (T.-Y.C.); toasty1041@gmail.com (T.-C.L.); sudiptasaha49@yahoo.co.in (S.S.); happydry@ms36.hinet.net (H.-Y.L.); yesyesweiwei@gmail.com (S.-W.C.); walice@adm.cgmh.org.tw (A.M.K.W.); 2 Healthy Aging Research Center, Chang Gung University, No. 259, Wen-Hwa 1st Road, Taoyuan 333, Taiwan; 3 School of Medicine, Chang Gung University, No. 259, Wen-Hwa 1st Road, Taoyuan 333, Taiwan; 4 The Zanvyl Krieger Mind/Brain Institute, Johns Hopkins University, 3400 N. Charles Street 338 Krieger Hall, Baltimore, MD 21218, USA; E-Mail: gomezramirezm@jhu.edu

**Keywords:** visual-tactile integration, direction of motion, congruency, haptic approach, tactile stimulator

## Abstract

Information obtained from multiple sensory modalities, such as vision and touch, is integrated to yield a holistic percept. As a haptic approach usually involves cross-modal sensory experiences, it is necessary to develop an apparatus that can characterize how a biological system integrates visual-tactile sensory information as well as how a robotic device infers object information emanating from both vision and touch. In the present study, we develop a novel visual-tactile cross-modal integration stimulator that consists of an LED panel to present visual stimuli and a tactile stimulator with three degrees of freedom that can present tactile motion stimuli with arbitrary motion direction, speed, and indentation depth in the skin. The apparatus can present cross-modal stimuli in which the spatial locations of visual and tactile stimulations are perfectly aligned. We presented visual-tactile stimuli in which the visual and tactile directions were either congruent or incongruent, and human observers reported the perceived visual direction of motion. Results showed that perceived direction of visual motion can be biased by the direction of tactile motion when visual signals are weakened. The results also showed that the visual-tactile motion integration follows the rule of temporal congruency of multi-modal inputs, a fundamental property known for cross-modal integration.

## 1. Introduction

For a biological system, perception often requires information emanating from sensors of multiple modalities, such as vision, touch and audition [1–3]. For example, the interaction between audition and vision determines perceived speech [4] and perceived timing of collision [5]. Similarly, sound can influence the perceived roughness of a touched surface [6] and can also affect perceived surface slant [7].

Touch and vision are similar in that both sensory signals derive from a sheet of sensor arrays, cutaneous receptors in the skin and photoreceptors in the retina. Indeed, touch and vision are intuitively integrated to yield a holistic percept of the environment around us [8,9]. It has been hypothesized that cross-modal integration is processed in cortical regions that receive both visual and tactile signals [10–12] and it is of interested to understand where and how in the brain this occurs. Recent human psychophysical experiments have shed some light on these questions. Moore *et al.* reported a close interaction between saccade directions and the processing of tactile motion [13] as well as motion after-effect transfer between touch and vision [14], implying a hard-wired connection between tactile and visual systems for motion processing. Bensmaia *et al.* [15] found that the perceived speed of tactile motion is influenced by the speed of a concurrent visual-motion stimulus, again indicating the existence of cross-modal integration between touch and vision. Blake *et al.* developed a visual sphere with visual ambiguity in its direction of rotation and results showed that touching the sphere disambiguates the visual percept [16]. Finally, Shore *et al.* investigated the temporal constraints of the visual-tactile crossmodal congruency effect in an experiment in which vibrotactile targets were presented to the index finger or thumb of either hand while visual distractors were presented from one of four possible locations. Participants made speeded discrimination responses regarding the spatial location of vibrotactile targets while ignoring the visual distractors. The results showed that the cross-modal effects were significant when visual and vibrotactile stimuli occurred within 100 ms. [17].

Visual-tactile motion integration has been explored using several apparatuses, with tactile-array stimulators comprising a matrix of linear motors or actuators being the most sophisticated devices [13–15]. The spatial-temporal indentation pattern from a population of independently moving motors can create simulated motion with arbitrary stimuli directions and contour, a property that is suitable for a variety of haptic experiments [18,19]. However, current array stimulators with a motor-array arrangement that is dense enough to exceed the innervation density of peripheral afferents in the human fingerpad (such as the 400-probe stimulator) [20] are expensive and bulky. Consequently, most researchers use rotator motors with one degree of freedom, in which a rotating object is touched and the subject reports the direction of rotation or discriminates the surface speed [16,21]. Although the use of rotator motors has been a well-established method in somatosensory research [22,23], the commonly used apparatuses with rotator motors are limited by having only one degree of freedom (restricting motion to two opposite directions such as clockwise and counterclockwise) and the inability to control the indentation depth into the finger. Moreover, spatially aligning the positions of visual and tactile stimuli is difficult as both video monitors and tactile stimulators occupy substantial space. Here, we develop a novel visual-tactile cross-modal integration apparatus that consists of a visual display for presenting visual stimuli and a tactile stimulator with three degrees of freedom for presenting tactile-motion stimuli in arbitrary directions, speeds, and indentation depths. Additionally, this apparatus can present cross-modal stimulation in which the spatial locations of visual and tactile stimuli are perfectly aligned. Tactile stimulus saliency can be modulated by controlling the indentation depth and visual stimulus saliency can be modulated by adjusting the level of superimposed noise presented on the visual display.

Using this apparatus, we presented visual-tactile motion stimuli in which the directions of motion were either congruent or incongruent between sensory modalities, and participants reported the perceived visual direction of motion. The magnitude of visual-tactile integration was then gauged by the degree to which the perceived visual direction was biased toward the tactile direction of motion. We hypothesize that the direction of perceived visual motion can be biased by the direction of tactile motion when visual signals are weakened. We also hypothesize that visual-tactile motion integration follows the rule of temporal congruency of multi-modal inputs: The effect of multi-sensory integration is most robust when sensory information from multiple modalities coincides [1,24,25]. Accordingly, we predicted that the integration effect would peak when visual and tactile stimuli were presented simultaneously.

## 2. Experimental Section

### 2.1. Development of the Tactile Motion Stimulator

**Figure 1.**
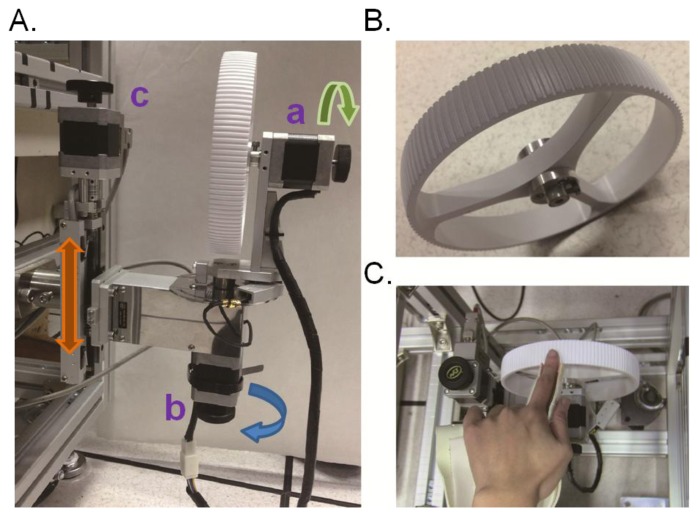
The tactile motion stimulator. (**A**) The three step motors (a, b and c) control each of the three degrees of freedom. (**B**) The stimulus drum. (**C**) The finger-hand holder.

To present tactile motion stimuli in specified directions, speeds, and indentation depths we developed a tactile motion stimulator (Figure 1(A)) with three degrees of freedom, controlled by three five-phase step motors (PK545-B, Oriental Motor Co. Ltd., Tokyo, Japan). Each step motor has a basic step angle of 0.72°, a precision that meets the needs of our experiments. One step motor rotates the stimulus drum for producing motion (Figure 1(A-a)). A second motor controls the arm for orienting the direction of stimulator motion (Figure 1(A-b)). The third step motor has a lead screw shaft (screw pitch, 1 mm; diameter, 9 mm; length, 150 mm) and translates rotational motion to linear motion, and adjusts the vertical excursion of the stimulator for controlling depth of indentation into the skin (Figure 1(A-c)). We used a programmable logic controller (PLC) to drive the step motors for the desired position and movement speed. The PLC was serially connected to a PC via an RS-232 port. In-house software using Matlab (MathWorks Inc., Natick, MA, USA) was developed to communicate with the PLC.

### 2.2. Stimulus Drum

The surface of the stimulus drum was made from a grating whose orientation was orthogonal to the direction of surface motion. Specifically, the stimulus drum (a truncated ball) had a diameter of 160 mm and was engraved with a square-wave grating of 3.9 mm wave length, 500 μm peak-to-peak amplitude, and a 0.4 duty cycle (ridge length/cycle length, Figure 1(B)). The drum was made of polyvinyl chloride and manufactured using Computer-Aided Design and Computer-Aided Manufacturing (CAD/CAM) to achieve high precision for these surface contours.

### 2.3. Finger-Hand Holder

The participant's left finger was supported by a finger-hand holder and was positioned volar-side-downward upon the upper surface of the stimulus drum (Figure 1(C)). The finger-hand holder was made from thermoplastic material so that it could fit the finger-hand anthropometric properties of each participant.

### 2.4. Experimental Setup for Visual-Tactile Experiment

**Figure 2.**
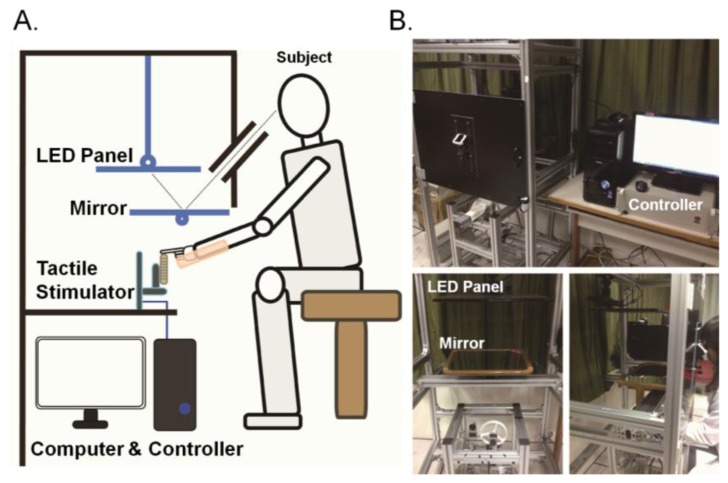
The apparatus for characterizing visual-tactile motion integration. (**A**) The schematic diagram of the setup that uses a mirror to achieve spatially aligned presentation of visual and tactile stimulation. (**B**) Three views of the apparatus and the control module.

We developed a setup that allows for spatially aligned presentation of visual and tactile stimulation (Figure 2(A,B)). The tactile stimulus was presented using the tactile stimulator and the visual stimulus via video displayed on the LED panel. A black board covered the entire setup and the participant was asked to place his left index finger on the stimulus drum to receive tactile stimulation and look at the screen through the small eyepiece fixed on the black cover to receive visual stimulation. The LED panel was placed above the tactile stimulator. The visual stimuli were videos of the subject's own index finger viewed through mirror reflection (inspired by a setup proposed by Ernst and Banks [26]), creating a visual experience as if the participant is looking at his own finger (Figure 2(A)). The assumption was that cross-modal integration would tend to occur when visual and tactile inputs were spatially matched. During the experiment, white noise was presented through earphones to avoid auditory cues that could arise from sound of the motors.

### 2.5. Generation of Visual Motion Stimuli

**Figure 3.**
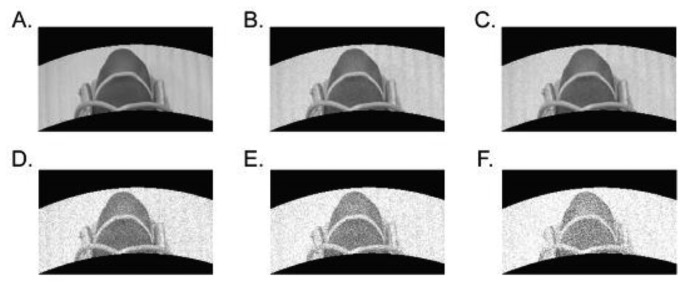
Snapshots from video clips with superimposition of different levels of Gaussian noise: Zero (**A**), 0.1 (**B**), 0.2 (**C**), 0.3 (**D**), 0.4 (**E**) and 0.5 (**F**). As can be seen in the example images, contour information is degraded as noise level increases.

We first video recorded a participant's own hand when the fingertip was presented with rightward (0°) or leftward (180°) motion. Use of the subject's own hand vivified the subsequent visual percepts. To eliminate possible cues elicited by the machine's shadow, the stimulus drum was visually presented through a specified aperture. The video was first transformed to gray scale, and different levels of Gaussian noise (Gaussian noise level, 0, 0.1, 0.2, 0.3, 0.4, or 0.5) were superimposed on each frame (Figure 3).

The assumption was that superimposition of noise would degrade the signal-to-noise ratio in the visual signals, allowing us to explore how visual-tactile integration may depend on visual signal certainty. The Gaussian noise level is defined by the ratio between the standard deviation of superimposed Gaussian noise and the largest luminance difference observed in the original image (Equation (1)):

(1) $$\text{Gaussian noise level}=\frac{\text{Standard deviation of superimposed noise}}{\text{Largest luminance difference in original image}}$$

### 2.6. Subjects

Ten volunteer subjects (five males, five females), ranging from 24 to 40 years of age, were paid for their participation. Five participated in the visual certainty experiment and seven in the temporal congruency experiment. Informed consent was obtained from each subject and the protocol was approved by the Institutional Review Board of Human Research of the Chang Gung Medical Foundation.

### 2.7. Visual Certainty Experiment

We performed two psychophysical experiments investigating the integration of visual and tactile signals in human observers. In the first experiment, subjects discriminated the direction of motion of visual stimuli when visual and tactile stimuli were simultaneously presented.

In a factorial design, the visual direction of motion was rightward (0°) or leftward (180°), tactile direction of motion was rightward (0°) or leftward (180°), and visual Gaussian noise level was zero, 0.1, 0.2, 0.3, 0.4 or 0.5. Both visual and tactile stimuli were defined by retinotopic (eye-centered) coordinates. Each stimulus condition was presented 10 times. The experiment was split into five blocks to allow subjects to rest so that each block contained 48 trials (2 visual directions × 2 tactile directions × 6 noise levels × 2 repetitions). The surface-motion speed of the tactile stimulus was 40 mm/s and its indention depth was 1 mm. In each trial, the visual-tactile motion was presented for 1 s and then the subject reported the direction of the visual stimulus by pressing one of two buttons on a computer mouse in a left-right two-alternative forced-choice design. The stimulus duration was the total indentation duration of the rotating drum, defined as the time from initial contact to the offset of indentation. Ramp-down period, defined as the time from initial contact to full indentation, and ramp-up period, defined as the time from full indentation to leave-off, lasted 0.15 s. The inter-trial-interval was 1.6 s. It was hypothesized that strength of visual-tactile motion integration can be gauged by the degree to which the perceived visual direction of motion is affected by the direction of tactile stimulus motion.

As a control experiment, we also performed a visual-only experiment in which the moving visual stimulus was presented as stated above, while the tactile stimulus was static (no surface motion), with a constant indentation of 1 mm. Each stimulus condition was presented 10 times. The experiment was split into five blocks and each block contained 24 trials (2 visual directions × 6 noise levels × 2 repetitions). Thus, we can compare task performance with and without tactile motion stimulation. We first performed the visual-only experiment and then the visual-tactile experiment.

### 2.8. Temporal Congruency Experiment

We then examined whether the results obtained in the previous experiment were compatible with the rule of temporal congruency of multi-modal inputs [1]. We performed a direction-congruency experiment with a variety of discrepancies in stimulus-onset latency. We hypothesized that the integration effect would peak when the onset of visual and tactile stimuli was simultaneous. We presented visual-tactile stimuli identical to those used in the visual certainty experiment, stimulus duration for each of visual and tactile stimuli was 0.5 s, and stimulus onset asynchrony (SOA), defined as the onset latency between the tactile and visual stimuli (Equation (2)), was −2, −1, −0.5, −0.25, 0, 0.25, 0.5, 1 or 2 s:

(2) $$\text{SOA}=L_{\text{tactile}}-L_{\text{visual}}$$

where *L_tactile_* and *L_visual_* are the onset latencies of tactile and visual stimuli, respectively. We first performed a pilot experiment to find the optimal visual noise level for individual subjects that could induce a specific level of visual uncertainty. The Gaussian noise level was chosen to induce accuracy range from 0.6 to 0.7 in the visual-only condition because the visual-tactile integration effect was observed robust within this Gaussian noise level in the visual uncertainty experiment. Each stimulus condition was repeated 10 times. The experiment was split into 10 blocks and each block contained 36 trials (2 visual directions × 2 tactile directions × 9 SOAs).

## 3. Results and Discussion

### 3.1. Results

#### 3.1.1. Visual Certainty Experiment

**Figure 4.**
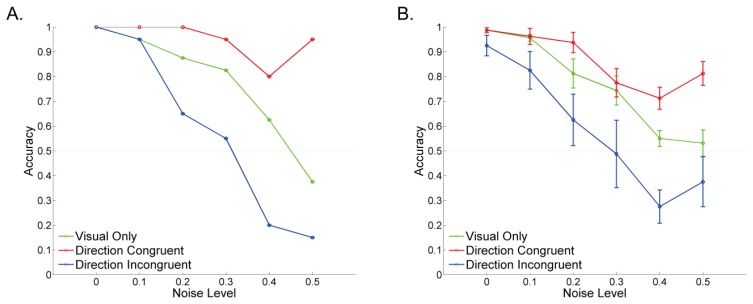
Accuracy in judging the direction of the visual-motion stimulus (left or right) as a function of visual noise level in visual only (green trace), direction congruent (red trace), and direction incongruent (blue trace) conditions. (**A**) Data obtained from a single subject. (**B**) Data averaged across subjects. Error bars indicate the standard error of mean. The results showed that perceived direction of visual motion was biased toward the direction of tactile motion, especially when visual noise level was high.

We used the visual-tactile apparatus to perform direction-congruency experiment with a variety of visual noise levels. In the visual only condition, we found that the probability of choosing the veridical direction of visual motion (accuracy) peaked at zero noise, monotonically decreased as noise levels increased, and finally reached chance level (accuracy = 0.5) at the maximum level of visual noise (Figure 4(A), green trace for data obtained from one subject; Figure 4(B), green trace for data averaged across subjects). The same trend was also found in direction congruent (Figure 4(A,B) red trace) and incongruent conditions (Figure 4(A,B) blue trace). Most importantly, compared with the visual only condition, accuracy was higher in congruent and lower in incongruent conditions. Specifically, perceived direction of visual motion was significantly biased toward the direction of tactile motion, indicating that visual and tactile motion information is integrated to yield a holistic percept (interaction effect in repeated-measured ANOVA, *p* < 0.05). Results indicate that the perceived visual direction is biased toward the tactile direction especially when visual noise level is high, providing evidence of visual-tactile integration.

#### 3.1.2. Temporal Congruency Experiment

**Figure 5.**
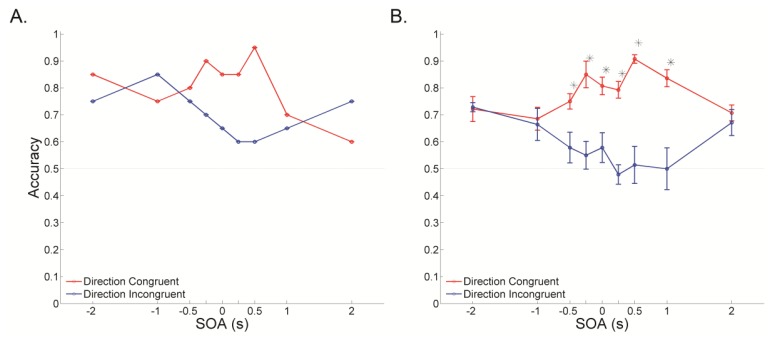
Accuracy as a function of stimulus onset asynchrony (SOA) in direction congruent (red trace) and direction incongruent (blue trace) conditions. For negative SOAs, the tactile stimulus preceded the visual stimulus; for positive SOAs, the tactile stimulus followed the visual stimulus. (**A**) Data obtained from a single subject. (**B**) Data averaged across subjects. The results showed that the degree to which perceived direction of visual motion was biased toward that of tactile motion peaked when SOA was close to zero. (*: *p* < 0.05 between the direction congruent and incongruent conditions).

We then examined whether visual-tactile motion integration follows the rule of temporal congruency of multi-modal inputs. We performed the temporal congruency experiment with several SOAs and a fixed visual noise level. Across SOAs, the strength of visual-tactile integration, gauged by the degree to which perceived direction of visual motion is biased toward the direction of tactile motion, peaked when the SOA was close to zero (simultaneous presentation) and gradually decreased as the SOA deviated away from zero (asynchronous presentation) (Figure 5(A), single subject; Figure 5(B), averaged across subjects). An interaction effect was observed using a repeated-measured ANOVA (*p* < 0.001). Post-hoc analysis using paired t-test showed that accuracy differed significantly between the congruent and incongruent conditions when SOAs were -0.5, -0.25, 0, 0.25, 0.5 and 1 s (*p* < 0.05). That is, visual-tactile integration peaks when visual and tactile stimuli are presented simultaneously, a finding that is compatible with the rule of temporal congruency of multi-modal inputs. Also, the rule of temporal congruency for visual-tactile motion integration has a relatively wide tolerance of SOA up to 1 s.

### 3.2. Discussion

Here, we introduce a novel visual-tactile cross-modal stimulation apparatus that allows for simultaneous presentation of visual and tactile motion stimuli at aligned spatial locations. This apparatus can present different combinations of visual and tactile stimuli, varying in direction and speed, while avoiding the physical constraint inherent in the one-degree-of-freedom rotator tactile stimulator. Most importantly, the signal-to-noise ratio of the visual stimulus can be modulated so that properties of visual-tactile motion integration can be more accurately characterized. To our knowledge, no previous stimulator apparatus has accomplished this. Other tactile motion stimulation apparatuses cannot align visual and tactile motion stimulation or precisely control the indentation depth of the tactile stimulus. Using this apparatus, several properties of cross-modal integration, including inverse effectiveness [27], temporal congruency [24], and spatial congruency [28] can be examined. Furthermore, the direction and speed constraints underlying cross-modal motion integration can be systemically characterized.

Results indicate that perceived direction of visual motion can be biased toward the direction of tactile motion when visual signals are degraded by the superimposition of noise. Because the percept could be dominated by tactile inputs when visual signals are uncertain, this finding implies that visual dominance of visual-tactile integration is adaptive as the percept could be dominated by tactile inputs. It also highlights the importance of including the capability to adjust the signal saliency for each modality when developing cross-modal integration stimulation apparatuses [29]. Using this apparatus, visual saliency can be modulated by the magnitude of superimposed noise while tactile saliency can be adjusted by indentation depth or the dimension of the engraved texture on the stimulus drum. Indeed, Fetsch *et al.* found that the neural system employs an optimal strategy of weighting cues from each modality in proportion to cue reliability [30], indicating the use of optimal probabilistic computation in neural circuits. That is, a modality whose signals are more salient will tend to be weighted more highly, a property that is consistent with our findings. This computation can be accounted for by Bayesian inference or maximum-likehood [26,31–33].

The results in the present study indicated that visual-tactile motion integration follows the rule of temporal congruency of multi-modal inputs. The temporal congruency is functionally relevant in that information from different senses occurring at approximately the same time most likely come from the same physical event [25]. Butz *et al.* [34] investigated visual-tactile motion integration and observed that the range of SOA that yielded significant integration could span 1 s, which was similar to the findings in the present study. However, Shore *et al.* examined the effect of spatial attention on the judgment of the position of a vibrotactile stimulus and found that the integration effect was significant only when SOAs were within 100 ms [17]. One possibility to explain this discrepancy is the difference of task structure. The present study was similar to Butz'*s* study in that these two studies investigated visual-tactile motion integration while Shore *et al.* studied position discrimination. Another possibility is that the stimulus duration was relatively longer in the present study (500 ms) and in Butz's study (960ms) while that in Shore's study was 10 ms.

Human brain imaging studies have showed robust increases of blood oxygen level-dependent (BOLD) signals in the extrastriate visual cortex when human observers are presented with tactile stimulus. These areas include the middle temporal (MT) [16,35] and medial superior temporal (MST) [36] cortices that are well known for their specialized processing of visual motion. The present stimulus apparatus will offer a unique opportunity to perform neurophysiological studies to characterize the functional relevance of these tactile-related BOLD signals in visual association areas. Finally, the apparatus can present different combinations of visual and tactile stimuli, varying in direction and speed. Although we did not use this feature here, future studies will use the apparatus to examine the speed and direction constraints underlying visual-tactile motion integration.

A haptic approach is usually applied in multi-sensory scenarios. It is then of utmost importance to characterize how information is processed in biological systems to infer a holistic percept. Furthermore, this apparatus will help develop cross-modal inference algorithms to determine how robotic systems resolve conflicting visual and tactile sensory information in scenarios that could occur in the real world [37]. However, to date, no neurophysiological experiment using non-human primates has been performed to explore visual-tactile motion integration. The main reason for this lack of information is instrumental limitation. The apparatus developed in the present study could be used for computational, psychophysical, and neurophysiological studies.

## 4. Conclusions

The present study introduces the design and demonstrates the validity of a novel visual-tactile motion integration apparatus that consists of a video display and tactile stimulator with three degrees of freedom. Using this apparatus, we showed that visual direction of motion is biased by the tactile direction of motion when visual signals are weakened. Additionally, the visual-tactile motion integration follows the rule of temporal congruency of multi-modal inputs. Further studies will be able to use this apparatus to investigate cross-modal motion integration mechanisms.
